# Supplementary material for: Nitrosyl-Heme and Heme Iron Intake from Processed Meats in Subjects from the EPIC-Spain Cohort
Source: Nutrients. 2024 Mar 18;16(6):878. doi: 10.3390/nu16060878 (PMC10975172; doi:10.3390/nu16060878)
Supplement: Supplementary file 1 [file nutrients-16-00878-s001.zip › nutrients-2911782-supplementary.pdf]

Nitrosyl-Heme and Heme Iron Intake from Processed Meats in Subjects from the EPIC-Spain Cohort

Lucía Rizzolo-Brime [1,2], Andreu Farran-Codina [3], Ricard Bou [4], Leila Luján-Barroso [1,2,5], Jose Ramón Quirós [6], Pilar Amiano [7,8,9], María José Sánchez [10,11,12], Miguel Rodríguez-Barranco [10,11], Marcela Guevara [12,13,14], Conchi Moreno-Iribas [12,13,14], Alba Gasque [13], María-Dolores Chirlaque [7,15,16], Sandra M. Colorado-Yohar [7,15,17], José María Huerta Castaño [7,15], Antonio Agudo [1,2] and Paula Jakszyn [1,18,]*

1 Unit of Nutrition and Cancer, Catalan Institute of Oncology—ICO, 08908 L'Hospitalet de Llobregat, Barcelona, Spain; lrizzolo@idibell.cat (L.R.-B.); llujan@iconcologia.net (L.L.-B.); a.agudo@iconcologia.net (A.A.)
2 Nutrition and Cancer Group, Epidemiology, Public Health, Cancer Prevention and Palliative Care Program, Bellvitge Biomedical Research Institute—IDIBELL, 08908 L'Hospitalet de Llobregat, Barcelona, Spain
3 Department of Nutrition, Food Science and Gastronomy, Faculty of Pharmacy, Institute of Nutrition and Food Safety (INSA-UB), University of Barcelona, Campus de l'Alimentació de Torribera, Av. Prat de la Riba 171, 08921 Santa Coloma de Gramenet, Barcelona, Spain; afarran@ub.edu
4 Food Safety and Functionality Program, Institute of Agrifood Research and Technology (IRTA), Finca Camps i Armet s/n, 17121 Monells, Girona, Spain; ricard.bou@irta.cat
5 Department of Public Health, Mental Health and Perinatal Nursing, Public Health, Mental Health and Perinatal Nursing, Universitat de Barcelona, Carrer de la Feixa Llarga s/n, 08907 L'Hospitalet de Llobregat, Barcelona, Spain
6 Public Health Directorate, 33001 Asturias, Spain; joseramon.quirosgarcia@asturias.org
7 CIBER Epidemiology and Public Health CIBERESP ISCIII, 28041 Madrid, Spain; p-amiano@euskadi.eus (P.A.); mdolores.chirlaque@carm.es (M.-D.C.); scyohar@gmail.com (S.M.C.-Y.); jmhuerta.carm@gmail.com (J.M.H.C.)
8 Ministry of Health of the Basque Government, Sub Directorate for Public Health and Addictions of Gipuzkoa, 20013 San Sebastian, Guipúzcoa, Spain
9 Biodonostia Health Research Institute, Epidemiology of Chronic and Communicable Diseases Group, 20014 San Sebastian, Guipúzcoa, Spain
10 Escuela Andaluza de Salud Pública (EASP), 18011 Granada, Granada, Spain; mariajose.sanchez.easp@juntadeandalucia.es (M.J.S.); miguel.rodriguez.barranco.easp@juntadeandalucia.es (M.R.-B.)
11 Instituto de Investigación Biosanitaria Ibs.GRANADA, 18012 Granada, Granada, Spain
12 Centro de Investigación Biomédica en Red de Epidemiología y Salud Pública (CIBERESP), 28029 Madrid, Spain; mp.guevara.eslava@navarra.es (M.G.); mc.moreno.iribas@navarra.es (C.M.-I.)
13 Instituto de Salud Pública y Laboral de Navarra, 31003 Pamplona, Navarre, Spain; alba.gasque.satrustegui@navarra.es
14 Navarra Institute for Health Research (IdiSNA), 31008 Pamplona, Navarre, Spain
15 Department of Epidemiology, Murcia Regional Health Council-IMIB, 30008 Murcia, Murcia, Spain
16 Social-Health Department, Murcia University, 30008 Murcia, Murcia, Spain
17 Research Group on Demography and Health, National Faculty of Public Health, University of Antioquia, Medellín 050010, Colombia
18 Blanquerna School of Health Sciences, Ramon Llull University, 08022 Barcelona, Catalonia, Spain
* Correspondence: paujak@iconcologia.net

Supplementary Table S1. All meat derivatives mean intake (g/d) from EPIC-Spain cohort and their levels of nitrosyl-heme and heme iron content measured by HPLC method

Items	Mean intake (g/d)	SD	NOheme* (µg/g)**	Heme iron (µg/g)**
Jamón serrano	5.30	9.8	39	41
Jamón cocido / jamón de York	5.04	9.6	22	27
Albóndigas de carne sin especificar, caseras	2.62	6.8	0	105
Croquetas de carne sin especificar	1.70	4.8	53	53
Chorizo curado	1.67	5.9	1	1
Tocino	1.33	3.2	1	1
Panceta de cerdo	1.11	3.7	6	17
Chorizo fresco oreado	1.01	5.0	31	29
Albóndigas de ternera	0.88	4.1	0	143
Salchichón	0.58	2.8	44	56
Hamburguesa de carne sin especificar, casera	0.58	3.7	0	128
Chistorra	0.46	2.7	30	35
Lacón	0.46	1.6	43	43
Frankfurt	0.41	2.4	80	82
Chorizo de Pamplona	0.34	2.6	38	37
Albóndigas de carne sin especificar	0.33	2.0	0	105
Salchicha fresca del país	0.32	2.0	0	71
Hamburguesa de ternera	0.30	2.7	0	136
Morcilla de cebolla	0.27	2.0	40	669
Morcilla murciana	0.22	1.5	51	281
Mortadela	0.20	2.3	14	19
Paté / Fuagrás / Foie-gras	0.18	1.4	97	97
Morcilla de arroz	0.16	2.4	16	555
Bacon	0.16	1.4	21	30
Albóndigas de cerdo	0.13	1.5	0	87
Longaniza de payés / llonganissa de pagés	0.13	1.1	29	45
Chóped / Chopped	0.12	1.6	21	31
Morcilla asturiana	0.12	0.5	242	440
Salchicha fresca murciana	0.12	1.2	0	65
Hamburguesa de pollo	0.10	1.4	0	82
Hamburguesa de carne sin especificar, bar/restaurante	0.09	0.0	0	128
Lomo embuchado	0.09	1.5	28	31
Fiambre de pavo	0.08	1.2	6	7
Sobrasada / sobrassada	0.07	1.1	28	28
Relleno	0.07	1.3	0	75
Longaniza imperial	0.05	0.9	42	42
Morcón	0.05	0.7	51	51
Salchicha de pollo	0.04	1.0	0	45
Chorizo tipo vela	0.02	0.4	64	69
Morcón blanco	0.02	0.6	26	26
Salami	0.02	0.6	27	28
Cabeza de jabalí	0.01	0.5	42	57
Sangrecilla	0.01	0.5	49	1554
Longaniza murciana / salchicha de pellizco	0.01	0.3	24	41
Longaniza seca	0.01	0.3	40	40
Cecina	0.01	0.4	105	183
Carne en lata	0.01	0.5	46	55
Hamburguesa de carne sin especificar	0.00	0.2	0	103
Bratwurst	0.00	0.0	2	29
Chicharrones	0.00	0.0	0	93

*NOheme; Nitrosyl-heme

**Data expressed as hemin (651.94g/mol)

Supplementary Table S2. Ranking of most consumed meat derivatives items that contribute most to total nitrosyl-heme intake in EPIC-Spain cohort

Food item	Contribution to total NOheme* intake= (µg/d)**
Jamón serrano	206.7
Jamón cocido / jamón de York	110.9
Chorizo curado	88.4
Frankfurt	33.0
Chorizo fresco oreado	31.2
Morcilla asturiana	29.0
Salchichón	25.7
Lacón	19.4
Paté / Fuagrás / Foie-gras	17.6
Chistorra	13.9
Chorizo de Pamplona	12.9
Morcilla murciana	11.9
Morcilla de cebolla	10.6
Panceta de cerdo	6.7
Longaniza de payés / llonganissa de pagés	3.7
Bacon	3.4
Mortadela	2.7
Morcilla de arroz	2.6
Chóped / Chopped	2.6
Lomo embuchado	2.6
Morcón	2.5
Longaniza imperial	2.3
Sobrasada / sobrassada	1.9
Croquetas de carne sin especificar	1.7
Tocino	1.3
Chorizo tipo Vela	1.2
Cecina	0.6
Sangrecilla	0.6
Cabeza de jabalí	0.6
Morcón blanco	0.5
Fiambre de pavo	0.5
Salami	0.5
Longaniza seca	0.4
Longaniza murciana /salchicha de pellizco	0.3
Carne en lata	0.3

*NOheme: nitrosyl-heme
**Data expressed as hemin (651.94g/mol)

Supplementary Table S3. Ranking of most consumed meat derivatives items that contribute most to total heme iron intake

Food item	Contribution to total heme iron intake= (µg/d)*
Albóndigas de carne sin especificar, caseras	275.0
Jamón serrano	217.3
Morcilla de cebolla	177.4
Jamón cocido / Jamón de York	136.1
Albóndigas de ternera	125.6
Morcilla de arroz	91.4
Chorizo curado	88.4
Hamburguesa de carne sin especificar, casera	74.4
Morcilla murciana	65.6
Morcilla asturiana	52.8
Hamburguesa de ternera	40.9
Albóndigas de carne sin especificar	34.1
Frankfurt	33.9
Salchichón	32.7
Chorizo fresco oreado	29.2
Salchicha fresca del país	22.9
Sangrecilla	20.3
Lacón	19.4
Panceta de cerdo	18.9
Paté / Fuagrás / Foie-gras	17.6
Chistorra	14.6
Chorizo de Pamplona	12.6
Hamburguesa de carne sin especificar, bar/restaurante	11.8
Albóndigas de cerdo	11.2
Hamburguesa de pollo	8.4
Salchicha fresca murciana	7.5
Longaniza de payés / llonganissa de pagés	5.2
Relleno	4.9
Bacon	4.8
Chóped / Chopped	3.8
Mortadela	3.7
Salchicha de pollo	3.3
Lomo embuchado	2.9
Morcón	2.5
Longaniza imperial	2.3
Sobrasada / sobrassada	1.9
Croquetas de carne sin especificar	1.7
Tocino	1.3
Chorizo tipo Vela	1.3
Cecina	1.1
Cabeza de jabalí	0.8
Fiambre de pavo	0.5
Salami	0.5
Longaniza murciana /salchicha de pellizco	0.5
Morcón blanco	0.5
Longaniza seca	0.4
Carne en lata	0.3
Hamburguesa de carne sin especificar	0.2
Chicharrones	0.0
Bratwurst	0.0

*Heme iron expressed as hemin (molecular weight = 651.94 g/mol)
